# Bidirectional modulation of hippocampal synaptic plasticity by Dopaminergic D4-receptors in the CA1 area of hippocampus

**DOI:** 10.1038/s41598-017-15917-1

**Published:** 2017-11-14

**Authors:** Sheeja Navakkode, Katherine C. M. Chew, Sabrina Jia Ning Tay, Qingshu Lin, Thomas Behnisch, Tuck Wah Soong

**Affiliations:** 1 Department of Physiology, Yong Loo Lin School of Medicine, National University of Singapore, Singapore, 117597 Singapore; 2 Neurobiology/Aging Program, Centre for Life Sciences, National University of Singapore, Singapore, 117456 Singapore; 3 The Institutes of Brain Science, The State Key Laboratory of Medical Neurobiology, and the Collaborative Innovation Center for Brain Science, Fudan University, 138 Yixueyuan Road, Shanghai, 200032 China; 4 National Neuroscience Institute, Singapore, 308433 Singapore

## Abstract

Long-term potentiation (LTP) is the persistent increase in the strength of the synapses. However, the neural networks would become saturated if there is only synaptic strenghthening. Synaptic weakening could be facilitated by active processes like long-term depression (LTD). Molecular mechanisms that facilitate the weakening of synapses and thereby stabilize the synapses are also important in learning and memory. Here we show that blockade of dopaminergic D4 receptors (D4R) promoted the formation of late-LTP and transformed early-LTP into late-LTP. This effect was dependent on protein synthesis, activation of NMDA-receptors and CaMKII. We also show that GABA_A_-receptor mediated mechanisms are involved in the enhancement of late-LTP. We could show that short-term plasticity and baseline synaptic transmission were unaffected by D4R inhibition. On the other hand, antagonizing D4R prevented both early and late forms of LTD, showing that activation of D4Rs triggered a dual function. Synaptic tagging experiments on LTD showed that D4Rs act as plasticity related proteins rather than the setting of synaptic tags. D4R activation by PD 168077 induced a slow-onset depression that was protein synthesis, NMDAR and CaMKII dependent. The D4 receptors, thus exert a bidirectional modulation of CA1 pyramidal neurons by restricting synaptic strengthening and facilitating synaptic weakening.

## Introduction

The dopaminergic system plays an important role in modulating learning and memory and synaptic plasticity^1^. The biological effects of dopamine are mediated by two kinds of receptor families, D1- and D2-like receptors. The D1-like dopamine receptors are positively coupled to adenylate cyclases (AC) and comprise D1 and D5-receptors, while D2-like receptors are negatively coupled to AC and comprise the D2, D3 and D4 receptors^2–4^. Many studies have been done to investigate the role of D1-like receptors in learning and memory, but the role of D2-like receptors has been much less studied. D4R, a member of the D2-like receptors, has been implicated in the pathophysiology of several psychiatric diseases like schizophrenia, attention-deficit hyperactivity disorder (ADHD), and autism which are characterized by cognitive deficits^5^. They are also present in brain regions critically involved in learning and memory like hippocampus and prefrontal cortex (PFC)^4^. While a majority of the studies on D4Rs were done in PFC, very few studies have focused on the hippocampus. Pharmacological inhibition of D4Rs in PFC was shown to be anxiolytic in rats, as assessed behaviorally by the elevated plus maze and shock probe burial tests, which demonstrated that D4Rs are involved in emotional learning^6^. D4R knockout mice exhibited reductions in behavioral responses to novelty and exhibited hyperexcitability of PFC pyramidal neurons via GABAergic transmission^7^. Zhong and Yan showed that the activation of D4 receptors did not affect the intrinsic excitability in PFC pyramidal neurons and parvalbumin positive interneurons^8^. D4R activation reduces AMPA receptor currents at potentiated synapses in hippocampal slices and AMPAR surface expression in cultured hippocampal neurons following chemical LTP^9^. D4Rs have been found to regulate working memory and other cognitive behaviors and they could modulate D1/D2- receptor ratio^10–12^. Moreover, D4Rs agonists were effective in restoring cognitive deficits in animal models^13,14^. Activation of D4R caused a significant reduction of excitatory transmission in acutely stressed animals and a marked increase of excitatory transmission in repeatedly stressed animals. As neural excitability is elevated by acute stress and reduced by repeated stress, D4Rs can act as a synaptic stabilizer in normal and pathological conditions^15^. Zhong and Yan showed that D4R activation decreased the frequency of spontaneous action potentials (sAPs) in PFC neurons, while in PFC parvalbumin positive (PV+) interneurons it caused a decrease of sAP frequency. This result suggests that D4Rs exert distinct effects on synaptically driven excitability in PFC pyramidal neurons and interneurons, which are differentially altered in neuropsychiatric disorders^8^. D4R has also been reported to play an important role in neuropsychiatric disorders involving working memory deficits such as ADHD and schizophrenia^11,16^.

Activity dependent bidirectional modifications of synaptic strength are essential for learning and memory. Long-term potentiation (LTP) and long-term depression (LTD) are considered to be the cellular correlates of learning and memory^17,18^. LTP is the persistent increase in the strength of the synapses, while LTD is the persistent decrease in the strength of synapses after high frequency and low frequency stimulation respectively^19,20^. The Bienenstock Cooper Monro (BCM) theory explains that the amplitude and direction of synaptic plasticity are determined by the synaptic modification threshold between LTP and LTD^21,22^. Nogo receptors have been reported to maintain this homoeostasis by restricting plasticity, and acting as a negative regulator of LTP^23,24^. Therefore, these long-lasting forms of synaptic plasticity require the activation of positive regulators that enhance memory and negative regulators that limit the plasticity. We hypothesized that D4Rs might limit plasticity as it is negatively coupled to adenylate cyclases. In PFC, D4Rs are known to exert a bidirectional role in the regulation of CaMKII activity, which is critically involved in synaptic plasticity and memory^25^. In the PFC slices with high neuronal activity, D4 agonists produced a potent reduction of CaMKII activity while in low neuronal activity state, it caused an increase of the CaMKII activity. Thus, D4R fine-tunes the activity of CaMKII in an activity dependent manner. This bidirectional regulation of CaMKII enables D4 receptors to affect multiple cellular functions^25^. This provides a unique mechanism for D4R to function as a homeostatic synaptic factor to stabilize/synchronize network activity. D4Rs also stabilize cortical excitability by exerting dual effects on AMPA receptor trafficking. In PFC, D4Rs caused either depression or potentiation of AMPA mediated synaptic transmission depending on whether their activity was higher or lower^26,27^. These effects of D4Rs was dependent on the D4Rs that mediate bidirectional regulation of CaMKII activity^26^.

Many forms of memory depend on the bidirectional regulation of synaptic plasticity and a balance between LTP and LTD is necessary for cognitive processes^28^. Hippocampal LTP is correlated with long-term memory particularly the reference memory, while LTD has a role in working memory^29^. Mallaret *et al*. had postulated earlier that persistent LTD might improve the ability to forget or it is an active process to remove or inhibit extra information and improve the processing of important information^28^. Long-lasting forms of LTP and LTD follows another cellular property called synaptic tagging and capture (STC), which explains how short-term forms of memory, can be converted to long-term memories in a time-dependent manner^30–33^. According to the STC hypothesis, induction of weak forms of LTP/LTD sets a synaptic tag, while induction of strong LTP/LTD not only sets a tag, but also synthesizes plasticit- related proteins (PRPs). The tags set due to weak events can capture the PRPs from the stronger input in a time-dependent manner^30,31,34^. It is known that CaMKII plays an important role in the setting of synaptic tags in LTP^35^. The bidirectional regulation of CaMKII by D4R also makes it a candidate for synaptic tagging and capture processes.

The D4Rs are also involved in depotentiation, which is a cellular mechanism for forgetting. The D4R null mice lack depotentiation of LTP by theta pulse stimuli^9,36^. They also showed that neuregulin induced LTP depotentiation is dependent on the activation of D4R^36^. Low frequency stimulation of temperoammonic inputs to CA1 can depotentiate CA1 Schaffer collateral LTP and it involves activation of D4Rs^37^. As synaptic weakening can be facilitated by LTD and depotentiation, it can serve as a mechanism to maintain synaptic stability.

As D4Rs are known to function as a homeostatic synaptic factor to stabilize network activity, we were interested to investigate how D4R exerts their effects in the CA1 region of hippocampal slices. Therefore, our main objective was to study D4R effects in different forms of plasticity like LTP and LTD and to further evaluate its role in cognitive processes. We present here that blockade of D4Rs facilitates late-LTP and transforms early-LTP into a late form of LTP. This reinforced LTP was found to be dependent on protein synthesis, NMDAR activation and CaMKII phosphorylation, in addition to GABA_A-_ receptor mediated mechanisms. Paired-pulse facilitation experiments with D4R antagonists indicated that short-term plasticity was not affected. We also showed that baseline synaptic transmission was unaffected by D4R inhibition. Interestingly, we found that D4R inhibition during late-LTD and early-LTD prevented both forms of LTD. We also showed that D4R inhibition blocks LTD by blocking the synthesis of PRPs rather than setting of synaptic tags. The activation of D4R induced a slow-onset depression which is protein- synthesis, NMDAR and CaMKII-dependent. In additon, D4R activation immediately after tetanization depotentiated late-LTP.

## Materials and Methods

### Field recording

All the experimental procedures were performed in accordance with guidelines and protocols approved by the National University of Singapore (NUS) Institutional Review Board and according to the guidelines of the Institutional Animal Care & Use Committee (IACUC), NUS, Singapore. We used 174 transverse hippocampal slices (400 μm) prepared from 87 male Wistar rats (5–7 weeks old). The rats were decapitated after anesthetization using CO_2_ and the brains were quickly isolated and placed into cold (2–4 °C) artificial cerebrospinal fluid (ACSF). Transverse hippocampal slices of 400 μm were prepared from both the right and left hippocampus using a manual tissue chopper. Slices were incubated in an interface chamber (Scientific systems design) at 32 °C, with a flow rate of ACSF at 0.82 ml/min and ACSF contained the following (in mM): 124 NaCl, 4.9 KCl, 1.2 KH_2_PO_4_, 2.0 MgSO_4_, 2.0 CaCl_2_, 24.6 NaHCO_3_, 10 d-glucose. The carbogen consumption was 18 l/h. One monopolar stainless steel electrode (5 MΩ; A-M Systems) was placed in the stratum radiatum of CA1 region for stimulating one synaptic input, S1, and in synaptic tagging experiments two electrodes (5 MΩ; A-M Systems) were positioned in the stratum radiatum of CA1 region for stimulating two separate and independent inputs S1 and S2 to a single neuronal population. For recording the field EPSPs, one electrode (5 MΩ; AM Systems) was placed in the CA1 dendritic layer and signals were amplified by a differential amplifier (Model 1700, AM Systems). The signals were digitized using a CED 1401 analog-to-digital converter and analyzed using PWIN software (Leibniz-Institute for Neurobiology, Magdeburg, Germany).

Slices were preincubated for 3 h, which is critical for reliable long-time recordings of late-LTP and late-LTD^38^. After the pre-incubation period of 3 hours, an input-output curve (stimulus strength vs fEPSP slope) was plotted. To set the test stimulus intensity, a fEPSP of 40% of its maximal slope was determined. For stimulation, biphasic constant-current pulses were used. Late-LTP was induced using three stimulus trains of 100 pulses [“strong” tetanus (STET), 100 Hz; duration, 0.2 ms/polarity; intertrain intervals, 10 min]. Early-LTP was induced using a weak tetanization (WTET) protocol consisting of one 100 Hz train and 21 biphasic constant-current pulses; pulse duration per half wave, 0.2 ms; stimulus intensity for STET and WTET, 40% of maximal field EPSP. Late-LTD was induced using a strong low-frequency stimulation (SLFS) protocol of 900 bursts [one burst consisted of three stimuli at 20 Hz, and the interburst interval was 1 s (i.e., f = 1 Hz; stimulus duration, 0.2 ms/half wave; total number of stimuli, 2700)]^32,38,39^. This stimulation pattern produced a stable late-LTD *in vitro* for ≥ 8 h. In experiments in which a weaker induction of LTD (early-LTD) was investigated, a weak low-frequency stimulation protocol (WLFS) was used consisting of 900 pulses at a frequency of 1 Hz, and pulse duration of 0.2 ms/half wave, with 900 total stimuli. The baseline was recorded for ≥ 30 min before LTP/LTD induction; four 0.2 Hz biphasic constant-current pulses (0.1 ms per polarity) were used for baseline recording and testing 1, 3, 5, 11, 15, 21, 25, and 30 min post-tetanus or 21, 25, and 30 min post-LFS and thereafter once every 5 min up to 3 h maximum.

### Pharmacology

The D4R antagonist L-745, 870 (Sigma) was first dissolved in dimethyl sulfoxide (DMSO) to make a stock solution and stored at 4 °C. This stock solution was then diluted in ACSF to a final concentration of 50 nM, ensuring that the final DMSO concentration did not exceed 0.1%. These concentrations were used as they did not affect basal synaptic transmission^33^. The D4R agonists, PD 168077 (Sigma) and Ro-10-5824 (Tocris), were both used at 0.1 µM and anisomycin (Sigma), a protein synthesis inhibitor, and KN93 (Sigma), a CaMKII inhibitor were used at a concentration of 25 µM and 1 µM respectively. PD 168077 (Sigma), Ro-10-5824 (Tocris), anisomycin and KN93 were dissolved in DMSO to make stock solutions and later were dissolved in ACSF. The NMDA antagonist, AP5 (Sigma) was dissolved in water and was used at 50 µM concentration.

### Statistics

The data were represented as mean ± SEM. The average values of the slope function of field EPSPs (millivolts per milliseconds) per time point and group were analyzed using the Wilcoxon signed rank test when compared within one group, or the Mann–Whitney *U* test when data were compared between groups; *p* < 0.05 was considered as statistically significantly different.

## Results

### D4 receptor blockade augments long-term potentiation

**Figure 1:**
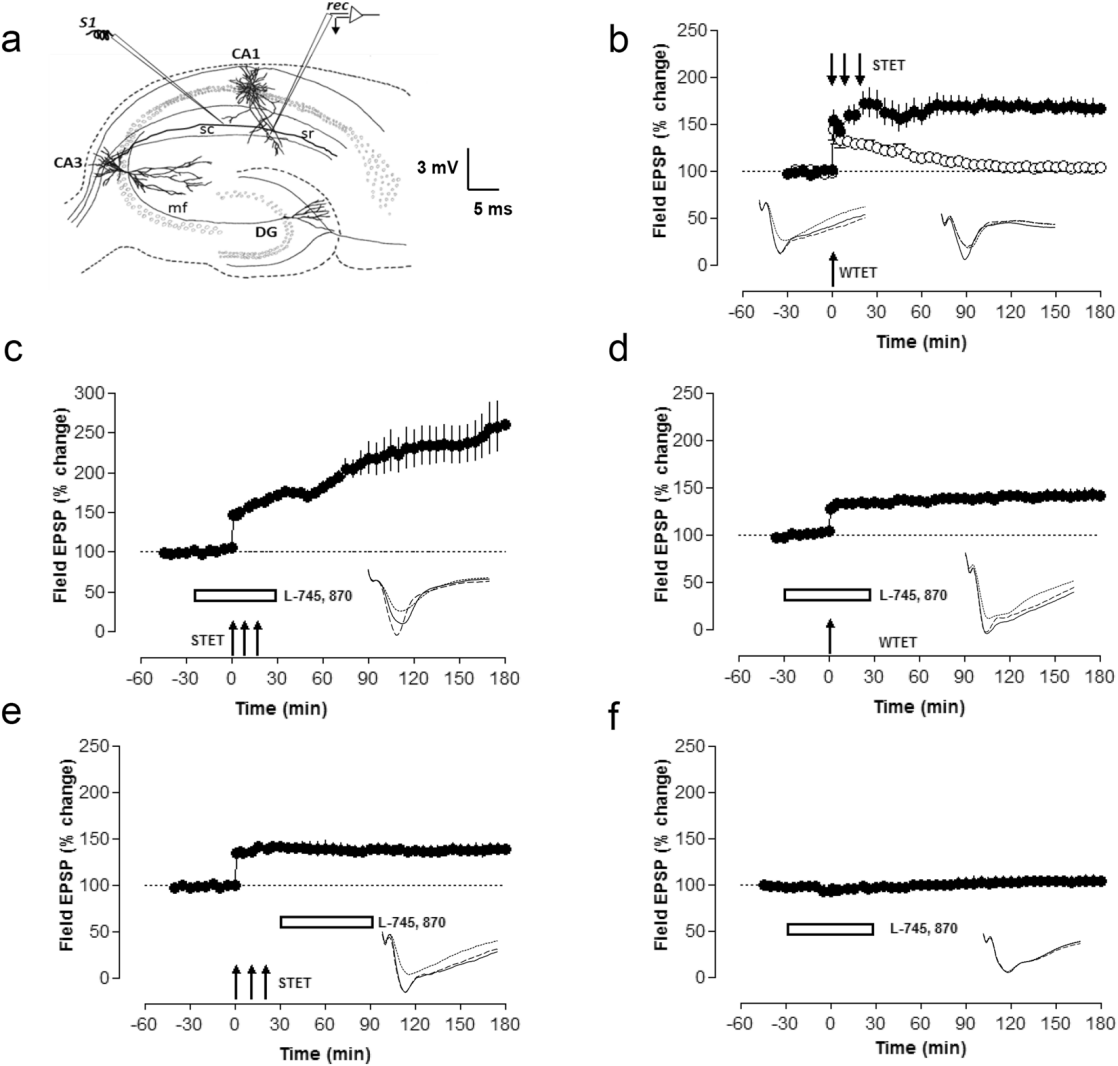
D4 receptor inhibition augments LTP. (**a**) A schematic representation of a transverse hippocampal slice showing the position of electrodes in the CA1 region of hippocampus. S1 represents a stimulation electrode used to stimulate a single neuronal population. ‘Rec’ represents a recording electrode. (**b**) Induction of late-LTP by a STET protocol resulted in a long-lasting LTP which was stable for 3 h (n = 9, filled circles). Induction of early-LTP using a WTET protocol resulted in weaker form of LTP which was stable for 90 min (n = 8, open circles). (**c**) When late-LTP was induced in presence of the D4R antagonist, L-745, 870 (50 nM) the fEPSPs increased to a level significantly higher than in control late-LTP (n = 7, filled circles). (**d**) A similar experiment as in (**c**) but with the induction of early-LTP instead of late-LTP (n = 8, filled circles) which resulted in the reinforcement of early-LTP to late-LTP. (**e**) Late-LTP was induced using a STET protocol; 30 min after the induction of late-LTP, L-745, 870 was applied for 60 min. L-745, 870 did not affect the maintenance of late-LTP (n = 6, filled circles). (**f**) L-745, 870 did not affect basal fEPSPs (n = 7, filled circles). Here after recording a stable baseline, L-745, 870 was applied for 60 min. Representative fEPSPs at −30 (dotted lines), +30 (dashed line) and 180 min (solid line). WTET represents weak tetanisation to induce early-LTP and STET represents strong tetanisation to induce late-LTP. Horizontal rectangular bars indicate the duration of drug application.

It has been reported earlier that D4-receptors (D4Rs) modulate LTP differentially in different regions of the hippocampus in freely moving animals^40^. Here we determined how the blockade of D4Rs affected different kinds of LTP in the CA1 region of hippocampal slices *in vitro*. A control late-LTP was induced using a strong tetanisation (STET) paradigm which resulted in a stable long-lasting LTP (Fig. 1b, filled circles). Statistically significant potentiation was seen until 3 h of recording (Wilcoxon test, p = 0.02). An early-LTP was induced using a weak tetanisation protocol (WTET) that resulted in a transient form of LTP that is statistically significant for 90 min (Fig. 1b, open circles). When the D4R antagonist, L-745, 870 was applied 30 min before and up to 30 min after the induction of late-LTP, the fEPSPs increased significantly (Fig. 1c, filled circles) when compared with the normal late-LTP (Fig. 1b, filled circles). Statistically significant LTP was observed until 3 h when compared with its own baseline (Wilcoxon test, p = 0.027) and when compared with control late-LTP, the synaptic potentials were significantly different from 120 min (U-test, p = 0.03) onwards until 180 min of recording (U-test, p = 0.03). As blockade of D4R during late-LTP resulted in enhancement of synaptic potentials, D4R might act to restrict plasticity. Therefore, we were interested to further investigate whether D4R blockade in weaker form of LTP like early-LTP would restrict plasticity. For this purpose, D4R antagonist, L-745, 870 was bath applied 30 min before and up to 30 min after WTET, and we observed that early-LTP was reinforced into late-LTP (Fig. 1d, filled circles). Statistically significant LTP was observed until 180 min of recording (Wilcoxon test, p = 0.005). In order to evaluate whether D4Rs exerted their effects during or after LTP induction, we applied L-745 870 also after the induction of late-LTP. Therefore, we repeated the same set of experiments as in Fig. 1c, but with the exception that the drug was applied 30 minutes after the induction of late-LTP (Fig. 1e, filled circles). Statistically significant potentiation was seen until 3 h of recording (Wilcoxon, test, p = 0.02). As control experiments, we also examined whether D4R blockade has any effect on basal synaptic transmission and our results showed that it has no effect on basal synaptic transmission (Fig. 1f, filled circles). Statistically significant potentiation was not observed throughout the time of recording and field EPSPs remained stable for 3 h.

### Properties of D4R inhibition mediated enhancement of early-LTP

**Figure 2:**
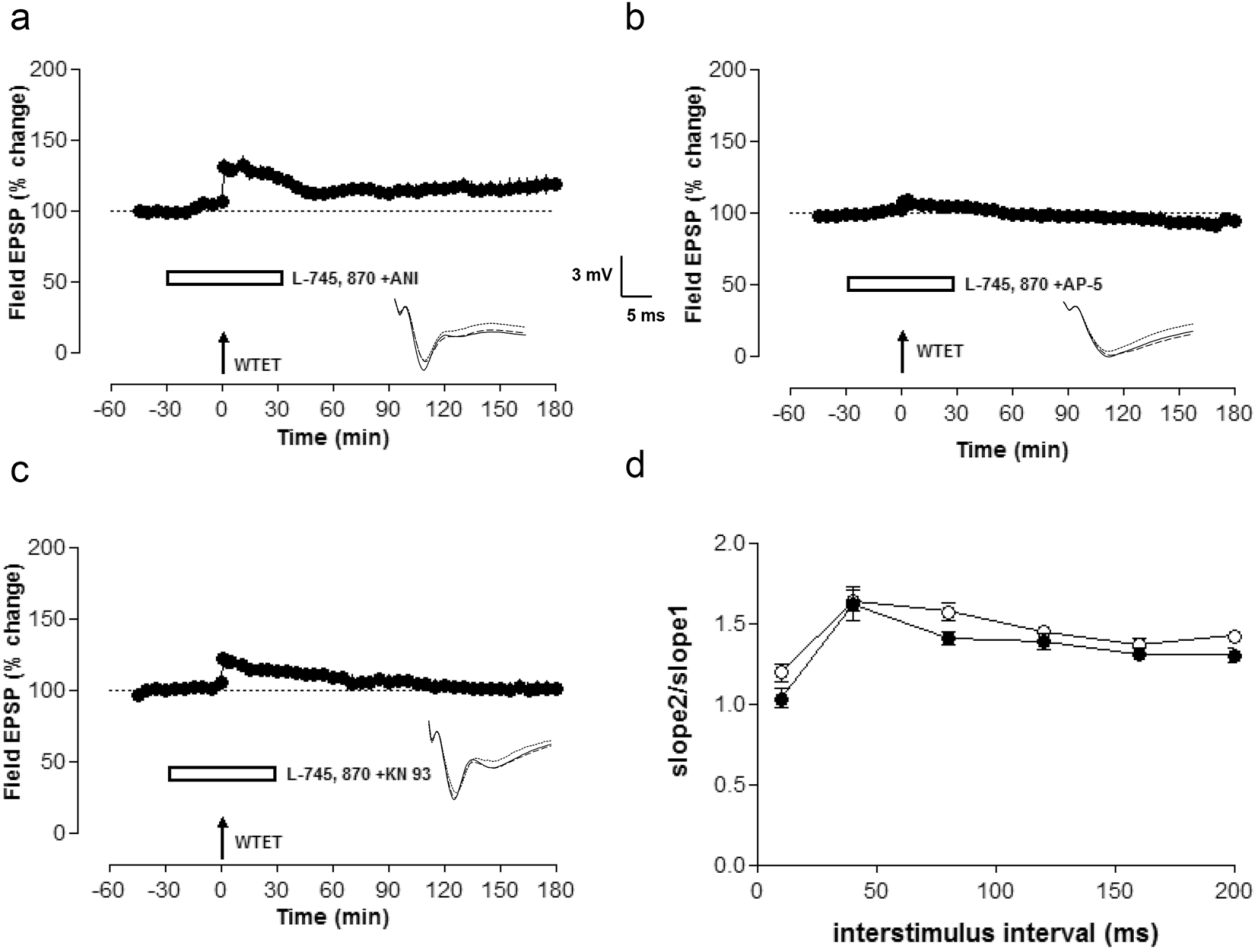
Properties of D4R inhibition mediated reinforcement of early-LTP. (**a**) In order to study the properties of the reinforced early-LTP by D4R inhibition, we used anisomycin, a reversible protein synthesis inhibitor. L-745, 870 was co applied with anisomycin for 60 min and a WTET was applied 30 min after the L-745, 870 application. Reinforcement of early-LTP into late-LTP was prevented by anisomycin (n = 7, filled circles). (**b**) The same experiment was repeated with the exception that (**b**) AP5 (**c**) and KN93 were used instead of anisomycin. The NMDA-receptor antagonist, AP5 blocked the induction of LTP (n = 7, filled circles). (**c**) The CaMKII inhibitor KN93 reduced the induction and prevented the reinforcement of LTP (n = 7, filled circles). (**d**) Paired pulse facilitation (PPF) in control (n = 7, filled circles) and L-745 870 (n = 7, open circles) treated slices did not show any significant difference.

As D4R inhibition reinforced early-LTP into late-LTP, we studied the properties of the reinforced early-LTP by D4R inhibition by using anisomycin, a protein synthesis inhibitor. Anisomycin and L-745, 870 were co-applied 30 min before and up to 30 min after the induction of early-LTP (Fig. 2a, filled circles). Statistically significant LTP was observed until 40 min (Wilcoxon test, p = 0.035) and from 45 min onwards. the field EPSPs reached the baseline levels. Next, in order to test whether the late-LTP observed after D4R blockade is dependent on NMDA-receptor activation, we did similar experiments as in Fig. 2a, but with AP5. AP5 completely blocked the induction and maintenance of LTP (Fig. 2b, filled circles). A statistically significant potentiation was not observed at any time points. Then we were interested to study whether CaMKII played a role in the reinforcement of early-LTP, as CaMKII is important for the maintenance of late-LTP^35,41,42^. When KN-93, a CaMKII inhibitor was co-applied with L-745, 870, a statistically significant LTP was observed until 35 min (Wilcoxon test, p = 0.01), and from 40 min onwards, the synaptic potentials returned to the baseline values (Fig. 2c, filled circles). Paired pulse facilitation experiments showed no significant difference between control (filled circles) and L-745, 870 treated slices (open circles) (Fig. 2d).

### The role of GABA_A_ receptors in D4R action

**Figure 3:**
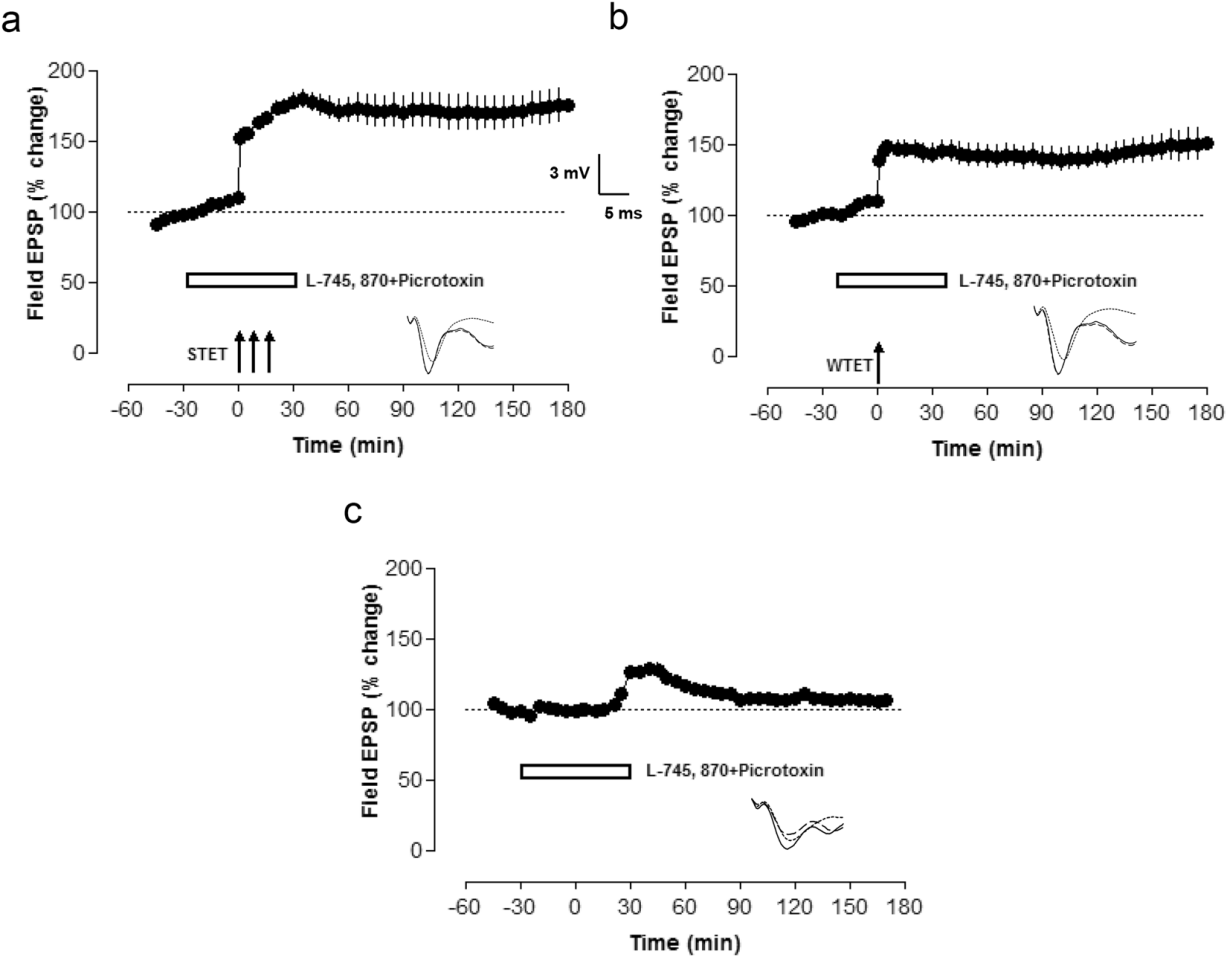
The role of GABA_A_ receptors in D4R action. (**a**) When picrotoxin, a GABA_A_ receptor antagonist was coapplied with L-745, 870 during the induction of late-LTP, the enhancement of LTP seen with L-745, 870 (as in Fig. 1c) was attenuated. (n = 6). (**b**) Similar experiments were repeated like in (**a**) with the exception that an early form of LTP was induced instead of late-LTP. When early-LTP was induced in presence of L-745, 870 and picrotoxin, it did not induce any significant changes in late-LTP (n = 6, filled circles). (**c**) To study whether picrotoxin when coapplied with L-745, 870 has any effect on baseline synaptic transmission, both drugs were coapplied for 60 min after recording a stable baseline for 30 min (n = 5, filled circles).

As D4R mRNA and proteins have been detected in GABAergic interneurons of the hippocampus, we wondered whether GABA_A_ receptors were involved in a D4R blockage-mediated enhancement of LTP^43–45^. Therefore, the GABA_A_ inhibitor, picrotoxin was co-applied with L-745, 870 thirty min before and until 30 min after the induction of late-LTP and picrotoxin reduced the enhancement of fEPSP potentiation but without blocking late-LTP (Fig. 3a, filled circles). The slope values were significantly different from baseline until 3 h (Wilcoxon test, p = 0.02) of recording and similar to control late-LTP values (Fig. 1b, filled circles). We also studied the role of GABA_A_ inhibition in the early-LTP reinforced by D4R blockade. Picrotoxin did not prevent the transformation of early-LTP into late-LTP (Fig. 3b, filled circles). The slope values remained statistically significant from 1 min until 3 h of recording when compared with its own baseline values (Wilcoxon test, p = 0.02). Then as control experiments, we studied whether L-745, 870 when coapplied with picrotoxin, has any effect on basal synaptic transmission. Picrotoxin induced a potentiation, from 40 min until 55 min (Wilcoxon test, p = 0.04) which came to the baseline within 20 min (Fig. 3c, filled circles).

### The role of D4R in long-term depression and synaptic tagging

**Figure 4:**
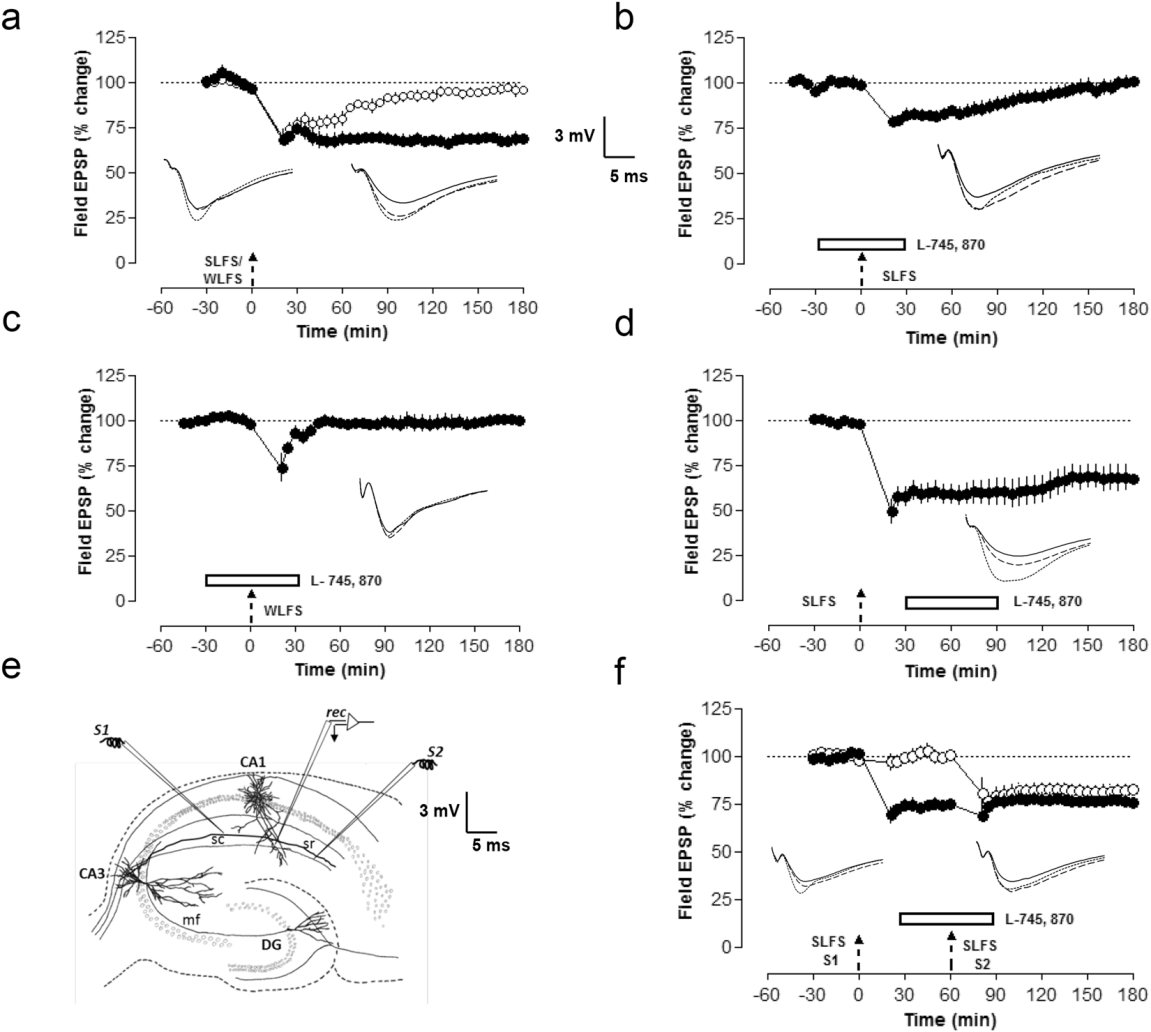
D4 receptors are critical for long-term depression. (**a**) Late-LTD can be induced by delivering SLFS, and an early-LTD can be induced using WLFS. SLFS induced a late-LTD which is stable for 3 h (filled circles, n = 8), and WLFS induced an LTD which is short lasting (open circles, n = 7). (**b**) L-745, 870 was applied 30 min before and 30 min after the induction of late-LTD after recording a stable baseline. L-745 870 blocked the late phase of LTD (n = 7, filled circles). (**c**) Next we studied the role of D4R inhibition during early-LTD. A similar experiment like (**b**) was repeated but with the induction of early-LTD (n = 6, filled circles). D4R inhibition blocked the maintenance of early-LTD. (**d**) In order to study whether D4R antagonist effect on LTD is activity dependent, L-745 870 was applied 30 min after the induction of late-LTD for 1 h. The D4R antagonist did not alter the maintenance of LTD which shows that D4R activity is not required for the maintenance phase of late-LTD (n = 6, filled circles). As early-LTD was also affected by D4R inhibition, we studied the role of D4R in synaptic tagging and capture. (**e**) Shows a schematic representation of a hippocampal slice with two stimulating electrodes in the CA1 area of hippocampal slice. S1 and S2 represent two stimulating electrodes to stimulate two independent synaptic inputs to a single neuronal population from where the recordings were made. ‘rec’ represents a recording electrode used to record the changes in synaptic activity. (**f**) After recording a stable baseline for 30 min, a SLFS was delivered to input S1. 30 min later L-745 870 was bath applied for 60 min, and 60 min after the induction of late-LTD in S1 another late-LTD was induced in synaptic input S2, but now in the presence of L-745, 870. Thus during the induction of late-LTD in S2 while D4Rs are blocked allowed the formation of synaptic tag and the capture of previously induced proteins transforming a declining fEPSP potentiation into a long-lasting one (n = 6, filled circles). SLFS-strong low frequency stimulation, WLFS-weak low frequency stimulation.

Next, we were interested to determine whether D4Rs have any role in LTD. Late-LTD or early-LTD was induced using strong low frequency stimulation (SLFS) paradigm and weak low frequency stimulation (WLFS) paradigm, respectively. Induction of late-LTD resulted in a long-lasting LTD which was stable for 3 h (Fig. 4a filled circles). Statistically significant LTD was observed from 21 min until 3 h of recording (Wilcoxon test, p = 0.02). The induction of early-LTD by WLFS resulted in a transient form of LTD which returned to the baseline values within 100 min (Wilcoxon test, p = 0.04) and then remained stable throughout the time of recording (Fig. 4a open circles). In order to study the role of D4Rs in LTD, L-745, 870 was bath applied 30 min before until 30 min after the induction of late-LTD and it blocked the maintenance of late-LTD (Fig. 4b, filled circles). Statistically significant LTD was observed only until 85 min (Wilcoxon test, p = 0.04). As late-LTD was blocked by a D4R antagonist, we next assessed the role of D4Rs in early form of LTD. Here a D4R antagonist was bath applied 30 min before and 30 min after the induction of early-LTD. Compared to the normal early-LTD, D4R antagonist application prevented early-LTD (Fig. 4c, filled circles). Statistically significant LTD was observed until 40 min (Wilcoxon test, p = 0.01) and then from 45 min onwards, it returned to baseline levels. In order to evaluate whether D4Rs exerted their effects during or after LTD induction, we applied L-745, 870 also after the induction of late-LTD. Therefore L-745, 870 was applied 30 min after late-LTD induction for an hour (Fig. 4d, filled circles). Statistically significant LTD was observed until 3 h of recording (Wilcoxon test, p = 0.027), which shows that like in LTP, D4R blockade has to occur during the time of induction of LTD to exert its effects. As early-LTD was also affected by D4R antagonists (Fig. 4c, filled circles), we studied the role of D4R in the setting of synaptic tags. To study the role of D4R in synaptic tagging, late-LTD was induced in synaptic input S1 (Fig. 4f, filled circles) and 30 min later L-745, 870 was applied for 60 min. 60 min after the induction of late-LTD in S1, a late-LTD was induced in S2 (Fig. 4f, open circles), in presence of a D4R antagonist. Here, although D4R inhibition prevents late-LTD, the late-LTD was not impaired most likely due to the formation of a synaptic tag by the SLFS and the capturing of plasticity relevant proteins formed in response to the first SLFS under drug free conditions.

### D4R activation induces synaptic depression

**Figure 5:**
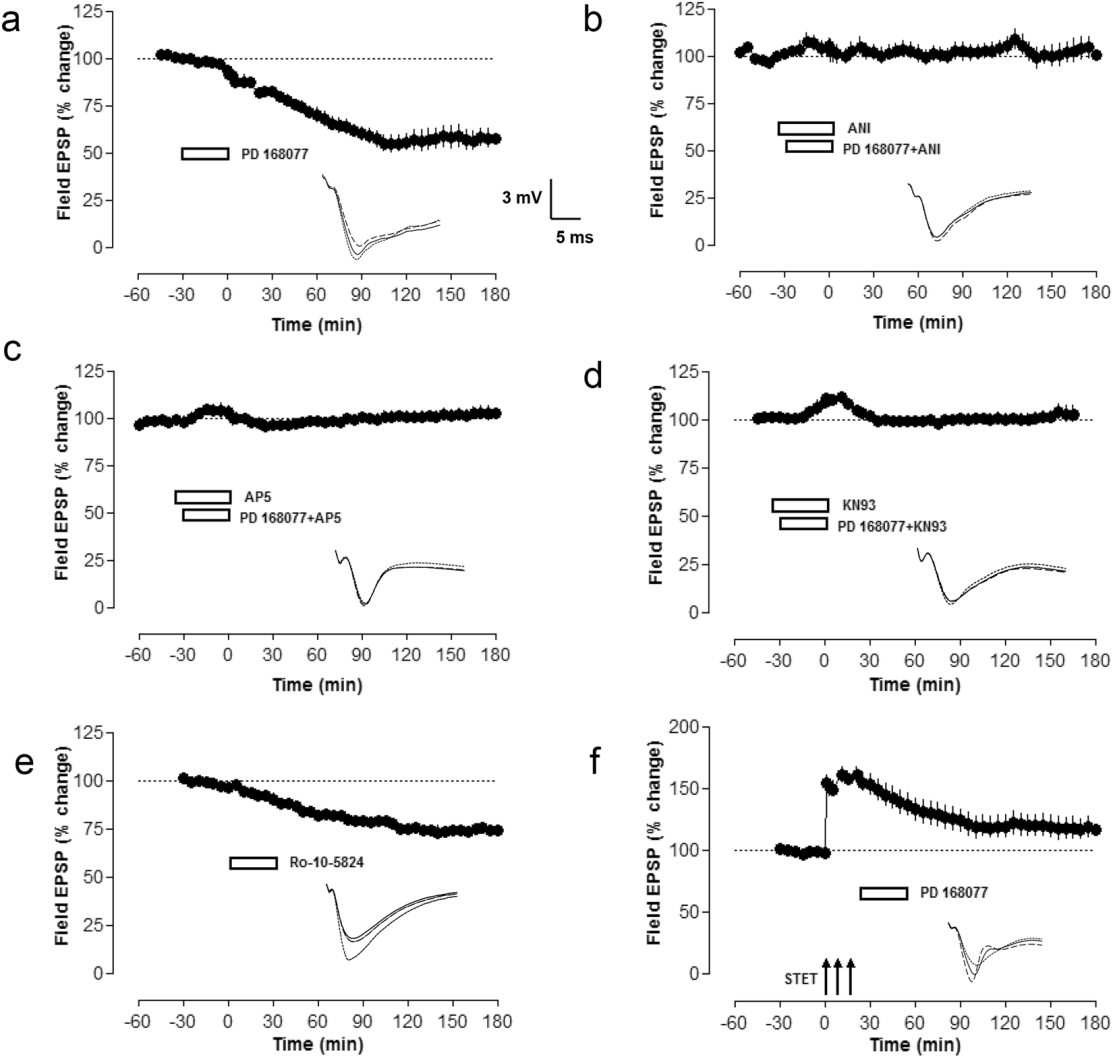
D4R activation induces synaptic depression. (**a**) The D4R agonist, PD 168077 induced a fEPSP depression that was stable for 3 h (n = 6, filled circles). PD 168077 was applied for 30 min after recording a stable baseline. (**b**) The same experiment was repeated as (**a**), but here Anisomycin was applied alone for 10 min before coapplication with PD 168077 for 30 min. Co application of PD 168077 with anisomycin prevented the fEPSP depression (n = 8, filled circles). (**c**) Here in order to study the role of NMDA-receptor activation, the experiment was done as in (b) but with AP5 instead of anisomycin and AP5 completely blocked the fEPSP depression (n = 6, filled circles). (**d**) The CaMKII inhibitor KN93 also prevented the fEPSP depression mediated by PD 168077 (n = 7, filled circles). (**e**) A D4R partial agonist, Ro-10-5824 was applied for 30 min and like PD, it induced a synaptic depression which was stable for 3 h (n = 7, filled circles). (**f**) The D4R agonist, PD 168077 was applied 5 min after the third tetanization (at 25 min) and was bath applied for 30 min. PD 168077 depotentiated late-LTP (n = 10).

As D4R blockade exerts differential effects in LTP and LTD, we were interested to study the role of D4R activation. Therefore, we used a D4R agonist, PD 168077, which by itself induced a depression of synaptic transmission (Fig. 5a, filled circles). Here after recording a stable baseline, PD 168077 was applied for 30 min. Statistically significant depression of fEPSPs was observed from min 25 (Wilcoxon test, p = 0.02) onwards until 3 h of recording. In order to decipher the underlying mechanism of D4R mediated fEPSP depression, the protein synthesis inhibitor, anisomycin was applied for 10 min alone before its coapplication with PD 168077 for 30 min (Fig. 5b, filled circles). A statistically significant fEPSP depression was not detected and the fEPSPs remained stable throughout the time period of recording (Wilcoxon test, p = 0.44). We repeated the same experiment as in Fig. 5b but used NMDA antagonist, AP5 and CaMKII- inhibitor, KN93 instead of anisomycin. AP5 prevented the induction and maintenance of D4R mediated synaptic depression (Fig. 5c, filled circles). The fEPSP slope values did not show a statistically significant depression and remained stable throughout the time period of recording (Wilcoxon test, p = 0.17). KN93 also blocked the synaptic depression (Fig. 5d, filled circles). The slope values were not statistically significantly different from min 1 until 3 h of recording (Wilcoxon test, p = 0.39). As our study is the first one showing depression of basal synaptic transmission via D4R activation in the hippocampus, it was important to confirm this finding with a D4R partial agonist. Thus, we confirmed our findings with PD 168077 (Fig. 5a), as well as using Ro-10-5824, which is a D4R partial agonist with high binding affinity. Ro-10-5824 induced a depression of synaptic transmission when applied for 30 min in a way similar to the effects of PD 168077 on baseline recordings (Fig. 5e filled circles). Statistically significant depression of fEPSPs was observed from 30 min (Wilcoxon test, p = 0.03) onwards until 3 h of recording. As D4Rs induced synaptic depression, we were interested to examine whether D4R activation after late-LTP would affect the maintenance of late-LTP. We could show that PD 168077 application 5 min after third high frequency stimulation (HFS) depotentiated late-LTP. The slope values were significantly different from 75 min (U-test, p = 0.04) onwards and until 3 h of recording when compared to late-LTP (Fig. 1b, filled circles).

## Discussion

The D4R specific modulators are known to improve cognitive deficits. Here, we determined how modulation of D4Rs affected synaptic transmission by examining their roles in LTP, LTD and depotentiation. We found that D4Rs restricted or fine-tuned plasticity depending on the nature of incoming information. In this way, D4R contributes to rescaling of synaptic transmission after induction of synaptic plasticity. If the D4Rs were blocked during the induction of late-LTP, which is dependent on transcription and translation, the synaptic potentials increased significantly. On the other hand, if they were blocked during the early-LTP, which was short lasting, early-LTP was reinforced into late-LTP.

Changes in neuronal activity like LTP and LTD can lead to changes in synaptic weight^46^. Recent evidences support the idea that the brain uses LTP-like phenomena to encode new information and LTD- like processes to inactivate old memories^47^. During LTP, the strength of the synapses increases, and if the synaptic strength does not stabilize, the neural networks would become saturated and all the information stored in the network will get degraded. Artificial saturation of LTP can lead to memory impairments^48^. Thus, saturation of the network must be prevented by preferential erasure of some previously encoded memories which is supported by the work from Xu *et al*.^49^ which showed that new spatial experiences could accelerate the decline of previously established LTP. Although a variety of molecules have been studied for their roles in encoding memory, fewer studies have been done on the roles of those molecules that restricts these synaptic weights^50–52^. We found a very specific role for D4R in restricting one form of plasticity like LTP, and facilitating LTD and synaptic depression and depotentiation. Kwon *et al*. have shown previously that D4R activation is known to depotentiate CA1 glutamatergic synapses and D4 mutants lack theta pulse stimulation mediated LTP depotentiation^9^. When the synapses are potentiated, some cellular activities should occur to weaken the synapses to prevent the synaptic saturation. D4R mediated enhancement of LTP was dependent on protein synthesis, NMDAR and CaMKII activation. The fast effect of anisomycin which we observed could be due to activation of p38MAPK^53^. The D4Rs might help to prevent saturation by facilitating LTD. This shows that modulating LTD, which is the weakening of synapses, is also an essential component of synaptic plasticity to maintain synaptic homeostasis.

Our results provide evidence that the modulation of D4R activity regulates the magnitude and direction of LTP and LTD. For example, it has been reported earlier that Nogo restricts the plasticity or it acts as a negative regulator of both functional and structural plasticity in mature neurons^24,54^ even though its inhibition did not affect short-term plasticity, LTD and basal synaptic transmission. D4R antagonist affected LTD without affecting the basal synaptic transmission. Our PPF experiments did not reveal any difference between control and L-745, 870 treated slices, confirming that short-term plasticity is not affected while D4R blockade prevented both early and late forms of LTD. Interestingly, we observed that interfering with D4R did not prevent the setting of synaptic tags, while it prevented synthesis of plasticity related proteins (PRPs). It is known that D4Rs bidirectionally modulate CaMKII phosphorylation and CaMKII represents a synaptic tag molecule that plays a pivotal role in the setting of LTP tags, but not LTD tags^35^. As D4R acts via a CaMKII dependent mechanism, our findings that D4R acts as a PRP and not a synaptic tag strengthen our previous finding that CaMKII acts only as a LTP specific tag and not an LTD specific tag molecule. Therefore D4R might help to balance the stability of neuronal networks by limiting LTP and facilitating LTD.

D4Rs facilitate inhibitory processes as they are coupled to inhibitory G-proteins and are known to inhibit the synthesis of adenylyl cyclases. Acivation of D4R also decreases L- type voltage gated calcium currents (VGCC) in PFC^55,56^ and they also couple to inwardly rectifying potassium channels^57^ through Gβγ-mediated mechanisms. Potassium channel modification also offers a powerful mechanism to fine-tune synaptic plasticity^58^. Ca^2+^ influx after synaptic activation activates the potassium channels that act to limit the amplitude of synaptic potentials and to reduce Ca^2+^ entry via NMDARs. Thus, during a high neuronal activity like LTP, D4Rs might act to limit or fine-tune the synapses by opening potassium channels. The D4 receptors are also known to interact with G protein-activated inwardly rectifying potassium (GIRK) channels, an important regulator of cellular excitability which reduces the firing rate of neurons. The GIRK currents are also activated by cells expressing D2 receptors in stable transfected cell lines^59^. This is supported by the findings that D4R knockout (KO) mice exhibited cortical hyper excitability^7^.

Our findings are supported by data reported from Kotecha *et al*. who showed that stimulation of D4Rs in CA1 area is known to depress NMDA receptor activity and excitatory synaptic transmission via the transactivation of platelet derived growth factor (PDGF)^60^. Activation of PDGFs is known to induce long lasting inhibition of NMDAR dependent EPSCs in the CA1 area^61^. Although the mechanism by which D4 receptors transactivate PDGF is not known, they showed that D4-receptors dynamically modulate synaptic transmission via Ca^2+^ dependent inactivation of NMDA receptors^60^. The D4R agonists caused a reversible decrease of the NMDA-receptor mediated current and reduced the amplitude of evoked EPSCs in prefrontal cortex^62^. In addition, Kohr and colleagues have shown that D4R negatively regulates NR2B containing NMDARs and LTP in stratum oriens^63^.

We have shown here that D4R agonists induce synaptic depression and it also depotentiates late-LTP. This is consistent with an earlier report that GIRK channels, which are known to interact with D4R are important for depotentiation, and GIRK null mice lack depotentiation, similar to the D4R KO mice^64^. As depotentiation also contributes to mechanisms that prevent the saturation of synaptic potentiation and increases the storage capacity of neuronal networks, D4Rs on the one hand decreases encoding and on the other hand facilitates inactivation/erasure of memories to prevent saturation of neuronal networks. D4Rs depotentiate LTP by regulating NMDA receptors^60,63^ and protein synthesis and it also reduces GABAergic transmission in PFC neurons. The D4Rs regulate both glutamatergic and GABAergic transmission which make a profound influence on networks innervated by the ventral tegmental area^27,65^. Activation of D4R has been shown to induce depression of AMPA EPSCs at high neuronal activity. For these experiments, Yuen *et*. *al* used PD 168077 at 40 µM concentration^26^ while Herwerth *et al*. used 0.1 μM PD 168077 without finding a depression of AMPA EPSCs in theCA1 area of hippocampus. The difference in the observed effects of PD 168077 on synaptic transmission in our experiments compared to one of Herwerth *et al*. could be based on the use of field potential recordings compared to the specific analysis of AMPA mediated currents by single cell recordings^63^.

The GABAergic transmission is known to be involved in the induction and maintenance of LTP^66,67^. As D4Rs are expressed in the parvalbumin positive interneurons, GABA_A_ receptors are likely to be involved in the D4R mediated modulation of synaptic transmission in CA1 neurons. However, we did not observe any alterations in the basal excitatory synaptic transmission but we noticed changes in the GABAergic system contributing to the enhancement of LTP. In order to maintain a delicate balance between excitation and inhibition, D4Rs might act on GABAergic system to maintain synaptic homeostasis. D4Rs in PFCs are known to decrease transport of GABA_A_ receptors^65^. In the hippocampus, we observed that inhibition of D4Rs increases the activity of GABA_A_ receptors since application of picrotoxin during the presence of a D4R antagonist prevented additional increase of synaptic strength. The localization of D4Rs in parvalbumin-positive interneurons, which are important for maintaining excitatory/inhibitory balance and cognitive functions, makes it an attractive target to study neuronal network activity.

Our results show that D4R activation modulates synaptic plasticity based on incoming information. If the incoming information is stronger then it restricts plasticity, but if it is weaker, then it facilitates plasticity. The D4Rs also play an important role in the weakening of synaptic strength and it is equally important for maintaining synaptic homeostasis. Thus D4Rs contribute to the stabilization of neuronal functions by fine tuning synaptic plasticity based on the incoming information. A better understanding of how D4Rs modulate synaptic plasticity might help to improve strategies for improving memory in neurodegenerative diseases such as Alzheimer’s disease.
